# Supplementary material for: Are carbon emissions trading and green financial instruments synergistic? -Comprehensive quantitative research based on content analysis
Source: PLoS One. 2024 Mar 7;19(3):e0298601. doi: 10.1371/journal.pone.0298601 (PMC10919601; doi:10.1371/journal.pone.0298601)
Supplement: S1 Data — (ZIP) [file pone.0298601.s001.zip › Data/地级市Z_F工作报告.zip!/╕ú╜¿/╧├├┼╩╨/└┤╫╘╣1⁄2╓┌║┼í╛┬φ┐╦╩2╛▌═°í┐_2004.docx]

各位代表：

　　现在，我代表市人民政府向大会作政府工作报告，请予审议，并请市政协委员和各位列席人员提出意见。

　　一、全面建设海湾型城市良好开局的一年

　　2003年，是全面建设海湾型城市的开局年，也是本届政府履行职责的第一年。面对繁重的改革发展稳定任务，面对突如其来的非典疫情和持续严重的干旱限电，我们在中共中央、国务院、中共福建省委、省政府和中共厦门市委的领导下，以邓小平理论和“三个代表”重要思想为指导，认真贯彻党的十六大和十六届三中全会精神，积极落实省委、省政府的各项决定，按照市委九届五次、六次、七次全会的部署，牢牢把握发展第一要务，团结奋进，攻坚克难，经过全市人民的共同努力，较好地完成了市十二届人大一次会议确定的各项任务。初步测算，全年完成国内生产总值760亿元，增长17.2%；地方财政收入73亿元，同口径增长19.1%；城镇登记失业率4.3%；城镇居民人均可支配收入和农民人均纯收入分别达到12 915元和5 172元，分别增长9.8%和9.5%。

　　这一年，我市国内生产总值增幅居全国同类型城市前列，规模以上工业总产值、社会消费品零售总额增幅创近年最高，外贸出口额突破百亿美元，港口集装箱吞吐量突破200万标箱并跻身全球30强，劳动力就业景气指数升至全国第五位，完成行政区划调整，抗击非典取得阶段性重大胜利，实现厦—金直航个案转通案，成功举办首届厦门国际马拉松赛和国际旅游节，荣获中国人居环境奖，摘取全国双拥模范城“五连冠”。

　　一年来，我们的主要工作是：

　　努力培植经济增长点，经济发展迈出新步伐。继续把工业发展作为支撑经济增长的主导力量，加强对工业企业的跟踪服务，确保戴尔计算机等一批产销大户高速增长，促成厦顺铝箔等一批增资项目顺利投产，完成厦门卷烟厂等一批老企业搬迁改造，集中扶持技术创新和创建名牌，抓好火炬高科技园区建设，高新技术企业产值增长37.6%，夏新、通士达、银鹭、惠尔康继厦华之后成为中国名牌，建发集团进入全国大企业集团百强，工业企业综合效益指数提高12个百分点。把招商引资作为积蓄发展后劲的重要手段，推出产业链、服务贸易、高新技术等六大领域招商政策，强化引资质量和项目储备，新引进安美光电等千万美元以上外资项目50个，承办的第七届“投洽会”规模与成效都超过往届。把开拓国际市场作为拉动经济增长的重要方式，降低进出口经营准入门槛，增加滞后退税的贴息贷款，积极争取当年退税指标61亿元，空港在国内首开“第五航权”，国际货运量升至全国第四位，海港增辟16条集装箱国际航线，推进口岸信息化和“大通关”工程，出口保持较快增长。把解决“三农”问题摆在更加突出的位置，集中扶持农业产业化龙头企业，农产品加工业产值增至种养业的3.7倍，扩大订单农业规模，建设如意果蔬等四个种苗基地和一批无公害农副产品基地，完成农村富余劳动力转移1.6万人，积极组织抗旱，基本解决旱区群众生产生活用水问题，促进了农民增收。把非典疫情带来的损失降到最低程度，及时采取阶段性税费减免、外来员工工资补助等措施，帮助交通运输、酒店、旅游等行业渡过难关，保证了第三产业迅速恢复增长。

　　实施城区扩展战略，海湾型城市建设全面推进。发挥规划的先导作用，修编城市总体规划，完成重点区域的规划调整，海湾型城市规划框架基本形成。坚持基础设施先行，环岛路陆域全线贯通，海湾大道部分路段、翔安大道、集美大道等岛外主干道开工，东通道等重点工程前期工作明显进展；海沧港区建设加快，完成猴屿西航道工程，开工建设厦门湾10万吨级航道二期，港口通航靠泊能力增强。积极为优化产业布局拓展空间，新增建设用地36平方公里，按照专业集中、区域连片、设施配套、服务健全的原则，整合形成了机械、电子、轻工、食品、石材等产业园区，引导企业向产业园区集聚。提升岛内城市建设水平，陆续展开莲坂等旧城改造，清理历年遗留的经营性房地产项目110个，腾出可用土地344万平方米，完成山体林相改造4 000亩，增加城市绿地222万平方米。修编海洋功能区划，加强无居民岛屿的保护管理，全面启动西海域清淤工程，生态效益开始显现。

　　加大各项改革力度，体制创新取得新突破。坚持以观念创新推进体制和机制创新，直面矛盾，稳步推进，出台了一系列促进改革发展的政策和措施。实施行政区划调整，行政区由7个调整为6个，资源、土地、人口等要素得到优化，部分事权下放，实现管理重心下移，调动了各区的积极性。加快国有企业改制，完成改制58家，分流安置7 500人，盘活资产28亿元。出台加快发展民营经济政策，鼓励和引导社会资本投资基础设施、公用事业和社会事业。改革工程建设管理体制，实行财政性基础设施项目建设阶段代业主招投标、经评审的最低价中标，加强标后管理，确保了工程质量，改善了建筑市场环境，节约了建设资金。改革征地拆迁机制，试行“一本城市规划，两级政府经营”。创新投融资体制，推出城市绿化、垃圾处理、海域保洁等项目向外资和社会资本开放，推行公交线路特许经营权招标试点，组建了水务集团，促进各种所有制经济共同发展。开展事业单位机构编制清理工作，启动事业单位改革。全面实施岛内“村改居”，完成村级换届选举。推行新一轮户籍制度改革。

　　万众一心抗击非典，确保人民生命安全和身体健康。我们依靠科学、依靠群众、依靠医务工作者，果断决策，精心组织，沉着应对。针对我市作为口岸，流动人口多、防控难度大的特点，较早制定了工作预案，严控陆海空关口，构筑预防网络，积极开展预防控制、医疗救治和卫生监督，千方百计组织急需物资、药品的生产和供应，切实做到早发现、早报告、早隔离、早治疗，成功治愈两例输入性病例，实现了无二代感染、无医护人员感染、无死亡病例的目标。按照做长期准备、建长效机制的要求，坚持“条块结合、以块为主”的属地化管理，广泛开展爱国卫生运动，加强科学防治宣传，增强市民健康意识，改进公共卫生工作。全市人民齐心协力、众志成城，确保了人心稳定、社会安定、经济发展，再次体现了厦门市民的良好素质，体现了社会各界同舟共济的协作精神。

　　坚持统筹协调发展，社会事业取得明显进步。把加快社会事业发展作为全面建设小康社会的重要举措，社会事业投入力度明显增强，已实施和安排的全社会投入超过55亿元。规划建设4所一级达标中学新校区，实行优质高中扩招，高中阶段教育普及率91.9%。改扩建10所初中学校，新增2 000名初中生如期入学。关心农村基础教育，解决2 600多名低困家庭学生就学。深化课程改革，素质教育得到推进。国家会计学院基本建成，完成3所成人高校合并迁建一期工程，职业教育和技能培训得到加强。结合纪念陈嘉庚先生创办集美学校90周年，全面修缮集美学村。增加公共卫生投入，扩建6家综合性医院，建立临床检验等4个重点医学中心，推进医疗机构集团化发展，迁建妇幼保健院，组建市疾控中心和卫生监督所，完善疾病控制和医疗救治体系，增加城乡卫生服务网点，社区医保服务站点增至58家。计划生育人口指标控制率和出生人口政策符合率连续十年居全省前列，拨出专款全面筛查伤残幼儿，进行早干预、早治疗，人口质量进一步提高。加快经营性文化产业的市场化步伐，探索新闻出版、广播影视业集约化经营的运作机制，着手组建广电集团，加快广电网络数字化改造。一批文艺作品在全国获奖。增加社区文体设施，广泛开展群众性文体活动，在第五届全国城市运动会上取得好成绩。开工新建射击射箭中心、同安体育馆等场馆。外事、对台、侨务、民族、宗教、气象、人防、方志、档案和民兵预备役等工作也取得新成就。

　　真心实意保障民生，城乡居民生活不断改善。始终把就业作为民生之本，全市所有街道、社区建立公共就业服务机构，重点帮扶“4050”人员、残疾人、复员军人和随军家属，全年城镇居民新增就业3.06万人。不断完善与经济发展水平相适应的社会保障体系，实现养老、医疗、失业、工伤、生育保险“五险合一”，调高最低工资标准，全市2.6万人享受城乡低保，各项社保覆盖率居全国前列。实行药品统一招标采购，药价平均下降23.8%，减轻群众就医负担7 700多万元。取消农业特产税，缓征农业税，清理违规收费，直接减轻农民负担4 000多万元。加大农村扶贫力度，重点帮扶31个经济薄弱村。完成提供廉租房、农村自来水供应等12件为民办实事项目，努力解决群众的困难。全面完成社区整合，社区服务功能进一步完善。举行城市抗震救灾联合演练。加强社会治安综合治理，开展安全生产、出租房安全、无证照燃气、食品安全卫生等专项整治。新辟、延伸15条公交线路，迁建蔬菜果品批发市场，试点推进“农改超”，启用小额支付卡，方便群众生活。建设青少年天文馆，增加老年人服务设施。帮助外来员工解决子女就学、工资拖欠、公交季卡乘车。妥善处理岛内油烟噪音、畜禽养殖等群众关注的问题，居民的生活环境进一步改善。

　　加强政府自身建设，行政水平进一步提高。深化行政审批制度改革，继续压减审批事项，减幅累计达62%，归并审批环节，建设项目审批缩短为45个工作日。加强对政府文件的合法性审查，组织法律知识学习，行政行为更加规范。深化效能建设和行风评议，统一公务员岗位津贴，严禁部门福利发放，勤政廉政得到加强。扩大政务公开范围，尝试政府常务会议向市民和媒体开放，建立政府新闻发布会制度，举行价格、规划等听证会，面向社会征集发展计策，政府决策的科学性和透明度提高。坚持对人民代表大会及其常委会负责并报告工作，自觉接受人大法律监督、工作监督和政协民主监督，主动与人大、政协沟通，建立重大事项通报机制。认真办理人大代表议案、建议和政协提案，办结率均达100%，人大代表和政协委员满意率分别达97.5%和94.7%。加强同各民主党派、工商联、无党派人士和各人民团体的联系，虚心听取各界人士的建议意见，民主渠道更为通畅。实行重点信访件跟踪督办，解决了一批信访疑难问题。

　　过去的一年，困难比预料的多，结果比预期的好，是1998年以来经济增长态势最好的一年，促进了经济和社会的协调发展，为“十五”后两年的发展赢得了主动。在实践中，我们坚持发展第一要务，始终保持强烈的忧患意识，把发展作为解决各类经济和社会问题的基础，盯住发展、谋划发展、致力发展、加快发展；坚持解放思想，开拓创新，用改革的思维、法治的规则、市场经济的手段，不断消除经济社会发展的体制性障碍；坚持正确处理改革、发展、稳定的关系，注意长远利益与眼前利益、整体利益与局部利益的协调，重视解决新时期不同利益群体之间的矛盾，形成加快发展的强大合力；坚持求真务实，深入基层调查研究、解决问题，力戒形式主义和虚报浮夸，认真履行政府职责，努力实现人民政府的承诺；坚持把群众是否满意作为政府工作的最终评判标准，注意倾听群众呼声，想方设法为群众排忧解难，实实在在提高全市人民的物质文化生活水平。成绩来之不易，这是全面贯彻落实中央、省各项方针政策和重大部署的结果，是中共厦门市委正确领导和市人大、政协支持监督的结果，是全市广大干部群众齐心协力、奋发图强的结果。在此，我谨代表市人民政府，向全市人民，向各民主党派、工商联、人民团体和各界爱国人士，向离退休老同志，向中央和省驻厦单位，向驻厦部队、武警和公安干警，向所有来厦的投资者和劳动者，向关心支持厦门的港澳同胞、台湾同胞、海外侨胞和国际友人，表示崇高的敬意和衷心的感谢!

　　各位代表，对照全市人民的期望，对比同类型城市的发展，政府工作还有很大差距，发展过程中仍然存在一些突出矛盾和问题，主要表现为：中心城市的辐射和带动作用不强。产业规模不大，研发创新能力较弱，产业链偏短；第三产业所占比重偏低，现代服务功能还不健全。一些制约发展的难题亟待破解，经济发展后劲不足。土地资源匮乏、电力能源不足对经济增长的制约增大，征地拆迁困难重重；深层次体制性障碍日趋显现，制约了经济特区优势的发挥；实际利用外资未取得突破性增长，民营经济的规模不大。社会发展存在的一些突出矛盾还未解决好。岛内岛外、城乡之间发展不平衡的问题还比较突出，农村富余劳动力转移压力较大；违法建设屡禁不止，安全生产仍然存在一些薄弱环节，群众对社会治安的满意率有所下降。公共服务与管理水平还不能适应社会发展需要。优质教育、医疗条件、文体设施尚不能满足群众需求；道路交通建设管理跟不上城市发展，部分路段出现拥堵现象；行政管理体制仍需进一步改革，少数干部责任心不强，不思进取、回避矛盾、怕负责任。对此，我们要高度重视，采取有效措施，认真加以解决。

　　二、以科学的发展观推进2004年工作

　　各位代表!党的十六届三中全会作出完善社会主义市场经济体制的决定，作为先行先试的经济特区必须按照中央要求，“增创新优势、走出新路子、实现新发展、办出新特色”。省委、省政府提出构筑海峡西岸经济区，要求厦门必须在“特、港、创、带、建”五个字上力求突破，更好地发挥龙头作用。市委先后作出了加快海湾型城市建设、加快区级经济发展、完善社会主义市场经济体制和增创经济社会发展新优势等一系列重要决定，全市人民加快发展的愿望更加强烈。与此同时，以区域经济板块为特色的竞争格局正在逐步形成，周边城市快速成长，与先进地区和同类型城市相比，我市发展的规模和后劲都有明显差距，形势逼人。我们必须把厦门的发展放在区域性、全国性乃至世界范围来审视，以前所未有的危机感和使命感，把增创经济特区新优势、提升城市综合竞争力，作为本届政府今后一段时期的努力方向，加快海湾型城市建设，努力把厦门建设成为我国东南沿海重要中心城市。

　　增创体制新优势。大力发展混合所有制经济，对现有国有企业，除国家另有规定外，原则上都要完成改制，通过引进战略投资者，优化资本结构；对已改制的企业，进一步完善法人治理结构，真正建立现代企业制度。完善市场体系，发挥市场在资源配置中的基础性作用，建设要素市场平台，促进各种生产要素合理流动和重组。健全“三农”工作机制，通过扶持龙头企业、发展订单农业，带动农民致富；完善“居住社区化、从业非农化、资产股份化、福利社保化”的配套政策，建立农民向二、三产业转移的机制，加快农村城市化。创新社会事业体制，对公益性部分，深化劳动人事、收入分配和社会保障制度改革，改善服务，增强活力；对经营性部分，通过改制转制，逐步推向市场，壮大实力。创新行政管理体制，切实把政府的经济管理职能，转移到主要为市场主体服务和创造公平竞争的良好环境上来。

　　增创科技新优势。全面提高产业的科技含量，密切科技与现有产业的结合，利用高新技术和先进适用技术改造传统产业，引进国内外先进的成熟技术在厦门组织生产，提高产业素质。构筑孵化转化基地，健全科技开发创新体系，发挥中试基地、重点实验室、企业孵化器和工程技术中心作用，吸引国内外最新科技成果来厦孵化、生产，使我市成为科技成果的重要转化基地。大力发展高新技术产业，在光电子、集成电路、数字视听、软件等领域，推动重大高新技术产业化，培育高新技术产业集群。提高信息化水平，立足服务全省，推动各类网络互联互通，提高覆盖率，构筑“数字厦门”的基本框架，促进信息技术在产业提升、经济发展和社会服务中的应用。

　　增创现代服务业新优势。结合服务贸易领域的开放、内地与港澳建立更紧密经贸关系安排的实施，加速形成区域性现代服务业中心。提升商贸物流业的竞争力，扩大保税区，试行港区联动，推动保税区向自由贸易区转型。大力发展电子商务、连锁经营、物流配送等现代流通。壮大旅游会展业规模，打好鼓浪屿、闽粤赣周边地区、金门旅游“三张牌”，努力形成以厦门为枢纽的旅游经济圈；充分利用会展设施，精心打造“投洽会”等大型会展品牌，进一步培育专业特色会展，努力成为全国重要的会展中心。拓展金融服务业，吸引更多金融机构来厦发展，在信用担保、信托投资、金融租赁等方面推动金融产品创新，大力发展资本市场，扩大金融业辐射范围，打造区域性金融服务中心。引进扶持中介服务业，形成强有力的中介服务优势。

　　增创港口新优势。深化“以港立市”战略，调整海空港规划，加快港区设施建设，完善集疏运体系，提升港口吞吐能力。增强辐射能力，加速形成通达各大洲主要港口的集装箱航运网络，增辟通达国内外的空中航线，完善公路、铁路快速交通网络，推动海陆空多式联运和转关运输。提高通关速度，加快口岸信息化，优化作业流程，推行诚信通关，强化配套服务，延伸平台业务，逐步向就地订舱报关报检过渡，建设通关便捷、服务周到、成本低廉的国际性中转港。

　　增创对台区位新优势。用足用好国家赋予的政策，推动厦门与台湾两地间市对市、港对港、行业对行业的交流合作。促进两岸产业对接，依托三大台商投资区，承接台湾新一轮产业转移，率先开放对台服务贸易，争取试行对台自由贸易政策。提升对台航运地位，扩大两岸试点直航成果，促进与台中、澎湖的海上和空中直航，拓展两岸客货运输模式。充分利用厦门与台湾的独特渊源，扩大民间往来，发挥涉台团体和台协等民间组织作用，采取灵活务实的方式，密切教育、卫生、文体等方面的双向交流，使厦门成为两岸实现直接、双向、全面“三通”的重要平台。

　　增创人居环境新优势。把环境指标置于与经济增长同等重要的位置，走高科技、高产出、低消耗、低排放的新型工业化道路，从源头上减少环境的压力。全面实施“蓝天、碧水、绿色、安宁、洁净”生态工程，创建生态社区，建设生态型城市。完善各类生活服务体系，维护良好的社会治安，保持稳定的社会秩序，营造明礼诚信、文明向上的社会氛围，创造温馨和谐、安全舒适的生活环境。

　　各位代表，本届政府将着眼长远，立足当前，扎实推进2004年工作。今年政府工作的总体要求是：以邓小平理论和“三个代表”重要思想为指导，认真贯彻党的十六大、十六届三中全会和省委七届六次全会精神，根据中央提出的“五个统筹”、“五个坚持”和“三十二字方针”，按照中共厦门市委九届七次全体(扩大)会议的部署，坚持以人为本，落实全面、协调、可持续的发展观，实施项目带动战略，全面推进海湾型城市建设，发挥中心城市的辐射带动作用，充分调动方方面面加快发展的积极性，增创经济特区发展的新优势，深化改革，扩大开放，促进投资，优化结构，保障民生，维护稳定，努力实现经济与社会持续快速协调健康发展。

　　2004年我市经济和社会的主要预期目标是：生产总值增长15%，地方财政收入同口径增长15%，城镇登记失业率控制在4.5%以内，城镇居民人均可支配收入和农民人均纯收入力争分别增长10%。实现今年目标，我们既要看到，世界经济回升，发达国家生产能力加快转移，我国继续实施积极财政政策和稳健货币政策，发展的宏观环境比较有利。同时也要看到，国际形势还存在许多不确定因素，台海局势面临复杂情况，国家出口退税政策的调整和我市偿债高峰期的到来，财政支出压力加大，土地、电力供应依然存在缺口，高致病性禽流感等周边疫情的影响不可低估。我们要把握机遇，正视困难，重点做好以下六个方面工作：

　　(一)狠抓项目带动，大力增强发展后劲

　　做大做强制造业。加强工业经济运行的分析调控，着力解决电力缺口等问题，及时帮助解决生产经营中遇到的困难。统筹使用工业发展专项资金，支持企业技术改造和开拓市场。加快古龙食口、腾龙树脂等项目建设，确保厦工工业园一期、林德气体二期等项目竣工投产，力促安美光电、太古飞机四期等工程早日动工，抓紧对二甲苯(PX)等项目前期工作。推动中药厂、电化厂等企业易地技改。推进光电子、软件等重点产业基地建设。鼓励企业争创驰名商标和名牌产品。培育壮大机械、电子、化工产业链，发展汽车零配件产业，促进贴片、模具中心建设，努力发展客车、芳烃、计算机等产业集群。推进火炬同集高新区和东部产业区建设，启动纳米技术、生物化学、海洋科学、材料技术研发中心项目，开展企业基础信息交换试点，提高科技对工业的贡献率。

　　大力发展现代服务业。继续引进国际知名零售、采购、物流企业，发展第三方物流，用政策鼓励大型商业企业到岛外布点，建设建材、汽车等大型专业市场。利用境外资本，推动城市商业银行增资扩股，改革农村信用社，提升地方金融机构竞争力。扶持发展银行卡产业，争取设立汽车金融公司、金融租赁公司，推动金融担保和期货业务的发展，引进或组建综合类证券机构和基金管理公司。实施鼓浪屿旅游票务制度改革，进一步加强与武夷山、闽西土楼等周边精品景区景点的互动，推动大陆居民经厦门赴金门旅游业务，密切与国际旅行商的合作，开拓海外客源市场，推进华天万豪等五星级酒店建设，提升旅游业服务水平。认真承办好“投洽会”，积极组织好礼品、石材、体育用品等专项会展。大力发展律师、会议、评估、咨询等中介服务业。

　　加快基础设施建设。重点项目安排继续向岛外倾斜，争取开工建设东通道，完成集美大道(孙厝—兑山段)、海湾大道(海沧段)和翔安大道一期工程，构筑岛外交通主干网。抓紧实现环岛路全线贯通，开工建设岛内南北快速通道，建成仙岳路、吕岭路、湖里大道，基本形成岛内网状连接。加强进出港航道建设、维护和管理，完善港区配套，改造疏港路，建成厦门湾10万吨级航道二期工程，加快建设海沧、嵩屿、东渡港区深水泊位和国际旅游客运码头。推动梧村汽车站、前场铁路货站和海沧铁路货场改扩建。启动杏林湾防洪排涝工程，扩建污水二厂和马巷水厂，动工建设翔安污水处理厂及部分污水管网、东部垃圾处理中心。加快杏林—高崎公铁大桥等项目的前期工作。加快电网改造，筹建燃气电厂等电力设施项目。

　　发展壮大民营经济。在市场准入、权益保护、生产要素配置和公共服务方面，为民营经济创造公平竞争的环境。落实鼓励政策，出台实施细则，吸引有实力的民营企业来厦发展，扶持有条件的民营企业做大做强。推进民营企业创业辅导、信息咨询服务体系建设，搭建民营企业与各类企业的合作平台。

　　(二)不断深化体制改革，为加快发展提供新动力

　　深化国企国资改革。加大国有企业改制力度，完善国有资本有进有退、合理流动机制，加快工业、商贸、建安等一般竞争性行业国有资本的退出；组建国有资产管理机构，整合国有资产投资公司和授权经营集团，完善国有资产授权经营制度。盘活社会事业国有资产，促进部分非经营性资产转为经营性资产。进一步完善财政性项目建设管理体制，实行项目前期工作代业主招投标试点，推行代建制。以明晰产权为重点，加快集体企业的股份制和股份合作制改革，发展多种形式的集体经济。

　　充分利用社会资本。加快投融资体制改革，鼓励社会资本进入法律法规未禁入的行业和领域，推出经营性社会事业、公用事业和基础设施项目对外开放，结合实行公用事业特许经营办法，加快公交、水务、燃气、垃圾处理等行业市场化进程，做好项目策划，吸引社会资本进入。充分利用壳资源，推动现有16家上市公司资产重组，促进有条件的企业在境内外上市。灵活运用银行扩大贷款利率浮动区间的政策，推进银企互动、银银合作，用足用好厦门金融存量资金。

　　激发事业单位活力。按照“脱钩、分类、放权、搞活”原则，深化事业单位改革，全面推行全员聘用制，实行职称评聘分开，开展学校校长、医院院长年薪制试点，扩大内部收入分配自主权。按照产业化和市场化方向，推进经营性事业单位转企改制，培育报业、广电等传媒产业，鼓励文艺表演团体走向市场，增强文化产业整体实力；整合优质医疗资源，面向周边及海外市场，拓展特色医疗服务。打破条块分割，理顺岛内外统一的道路、车辆、山林、水务等管理体制。

　　(三)进一步扩大开放，提高参与国内外竞争能力

　　务求引资新突破。全面落实六大领域招商政策，组建重大项目储备和策划中心，加强宣传推介，做深做细项目的投资分析，主动跟踪接洽国际相关企业。培养专业敬业的招商队伍，继续做好侨务工作，充分发挥海外华人华侨的作用，密切与台港澳的交流与合作，拓宽引资渠道。加强全市引资协调，建立共享的信息库，紧紧围绕园区的功能定位与专业特色，严格按产业规划和发展导向招商，提高引资成效。完善园区配套设施，调控土地增量，清理闲置土地，加快已批农转用地的征地平整，为招商提供成熟用地。改进项目跟踪负责制，缩短从签订合同到开工建设的时间。强化对现有外资企业的服务，发挥外商、台商协会作用，促进企业增资扩产。统一内外资招商机构，加强对内引资工作。

　　力争外贸大出大进。继续保持我市鼓励出口政策的稳定性，加强鼓励出口政策的宣传，引导企业充分运用出口信用保险政策，积极适应出口退税新机制，加快当年退税，兑现历年欠税。优化商品结构和出口方式，鼓励外贸企业走工贸型道路，由收购制改为代理制，实行出口加工区深加工结转，大力发展高附加值的加工贸易，培植出口优势产品，提高出口产品档次。加强地产品的宣传推介，组织企业到国外办展促销，巩固传统出口市场，开拓发达国家市场，有重点地拓展新兴市场，建立健全反倾销应诉快速反应机制，支持企业获得国际产品质量、环境管理和安全标准认证。鼓励有条件的企业“走出去”，到境外设立贸易网点、投资办厂和承揽工程。更加重视进口贸易，建设进口商品分拨中心。规范货运代理、船舶代理，推广应用口岸物流信息平台，继续整合通关系统，加强口岸监管业务协调，推动关检联验。

　　拓展对台经贸合作。积极跟踪台湾产业转移，促进厦门与台湾产业优势互补，大力吸引台湾光电企业和上市公司前来投资。拓展厦门—高雄海上试点直航新业务，推动货物直接通关入境。办好“台交会”，挖掘大嶝对台小额贸易市场的潜力，扩大对台贸易。进一步简化台胞入境手续，提供便捷、亲情服务，早日启动运转五年期台胞证受理中心，提高厦门作为台胞入出祖国大陆主要口岸的地位。

　　加强对内经济协作。密切关注长三角、珠三角的发展动态，主动参与经济分工与合作，争取两面受益。运用优惠政策，有针对性地招揽内地大企业来厦设立地区总部、研发中心和采购中心。加强厦漳泉专业化分工协作，深化与闽西南、闽粤赣及其他地区的区域经济合作，积极做好对口帮扶和山海协作，发挥外地驻厦机构的作用，继续关心支持内联企业的发展，加快内联合作项目建设。积极参与构筑闽西南快速交通网络，动工建设同安至安溪高等级公路，推进厦门—梅州海铁联运，加快厦漳跨海大桥、福厦铁路、龙厦铁路等前期工作。做好对西藏、宁夏、重庆等地区的对口支援。

　　(四)坚持统筹发展，促进城乡经济社会全面进步

　　规划重点向岛外转移。完成城市总体规划和土地利用总体规划修编的报批。着手进行各分区规划，以马銮湾、杏林湾和翔安区为重点，编制岛外市政、电网工程详规。完成本岛东部片区规划，制定旧城核心保护区法定图则。以项目为导向，搞好近期建设用地的控制性详规。完成全市海洋、环境功能区划和林地保护利用规划。

　　更加重视“三农”工作。优化农业结构，重点发展种苗业、无公害农业和生态观光农业，扶持农副产品加工，提高农业附加值。完善农产品批发市场，加快农业科技服务、市场信息、标准化和农产品质量安全体系建设，帮助拓宽销售渠道。加快农村城市化，实行部分镇改街，推进岛外村改居，引导村集体财产股份化经营。依法加强水资源管理，搞好沿海堤防和水库建设。完善防灾抗灾体系，采取强有力措施严控高致病性禽流感等疫情对我市的影响。逐步解决农村用水、用电和道路交通问题，争取两年内基本实现行政村道路硬化，整顿和发展农村客运市场。深化农村税费改革，完善涉农收费公示制度，严查乱收费、乱摊派行为，进一步减轻农民负担。

　　加快发展社会事业。统筹制定全市教育、卫生、文化、体育等社会事业发展规划。建设小区配套学校，扩建优质中学，扩大优质教育覆盖面。整合职教资源，组建职业技术学院，发展岛外中职教育，推进厦门城市大学建设。扶持民办学校，确保外来员工子女的义务教育。合理调整农村学校布局，增加农村教育投入，提高办学质量。继续共建厦大、集大，加快建设集美文教区，推进厦门理工学院、厦大医学院、集美大学和华文学院新校区建设，促成哈工大等高校来厦办学，规划筹建翔安文教区。加强综合性医院和特色专科医院建设。迁建疾病控制中心，新建紧急救援中心和传染病院。建立和完善计生工作新机制，进一步优化人口素质。筹建文化艺术中心，创办厦门文学院，推进地方文艺团体改革，办好第六届全国舞蹈比赛、海峡两岸歌仔戏艺术节和第二届钢琴节，举行城乡文艺调演，举办企业文化节，丰富群众文化生活，繁荣文化市场。开展全民健身运动，办好2004年夏新厦门国际马拉松赛。建成嘉庚体育馆和同安体育馆，筹建国际网球中心等场馆。争办国内外有影响的体育赛事。

　　争创全国文明城市。发动群众广泛参与创建“文明社区、文明村镇、文明行业”活动。贯彻《公民道德建设实施纲要》，弘扬科学精神和社会正气，加强社会公德、职业道德和家庭美德教育。加强科普工作，推进“四五”普法教育，广泛开展志愿者服务活动。加快推进社会信用体系建设，营造诚实守信的社会氛围。做好民族与宗教工作。增强国防意识，继续抓好民兵预备役工作，强化民防、海防管理，一如既往地支持驻厦部队、武警部队建设，进一步密切军政军民关系，依法妥善安置转业军人，提高双拥共建水平。

　　(五)坚持以人为本，提高人民群众生活水平

　　继续改善人居环境。以争创“联合国人居奖”为契机，提升城市建设和管理水平。认真实施环保条例，加强对重点污染源的监控治理，严格控制扬尘和油烟污染，治理机动车尾气，推广使用清洁能源。继续关闭一批采矿点，整治湖边、上李水库和岛内裸露山体，实施筼筜湖清淤及截污工程，继续西海域清淤工程。落实园林绿化责任制，增建绿地350万平方米，开工建设海湾、马銮湾、忠仑等8个公园，实施万亩林相改造和生态林示范工程，改良海岸防护林带，种植红树林，保护滩涂湿地。完善市容卫生考评机制，推进垃圾分类收集，治理厨余泔水污染。加快旧城旧村改造，出台鼓励政策，促进岛外房地产业发展。全面实施农村改水改厕，基本实现农村生活供水管网化，新建300个水冲式公厕和镇街的垃圾处理场，改善村容村貌。大力发展公共交通，规范停车秩序，提升交通管理智能化水平，保持城市交通畅通。

　　努力扩大就业规模。把新增就业人数和控制失业率作为重要调控目标，力争实现新增城镇就业2万人，转移农村富余劳动力1.5万人。加大再就业投入，健全市、区两级就业服务中心，完善社区公共就业服务和信息网络，建设同安、集美、海沧人力资源市场。鼓励未升学的初高中毕业生继续接受职业教育。加大技能培训力度，提高本市居民就业能力。通过实行社保和工资补贴及购买公益性岗位等办法，对“4050”、困难职工和随军家属实施再就业援助。培育劳务派遣组织，设立小额贷款担保基金，鼓励农民外出务工和劳务输出，促进农民就业和创业。加强劳动监察，完善劳动关系三方协调机制，规范企业用工和工资支付，遏制恶意拖欠工资、工程款等行为。

　　健全社会保障体系。扩大基本养老保险覆盖面，出台事业单位养老保险办法，逐步提高外来员工缴交比例与缴费标准，完善灵活就业人员的社保政策。落实最低生活保障办法。健全城镇职工基本医疗保险制度，增加社区卫生服务机构，开设药店医保定点服务。完善农村社保、合作医疗制度，积极发展商业养老、医疗保险。建立失地农民和转产渔民基本养老保险制度。整合社会互助、医疗、教育、住房、法律等社会救助资源，加强对流浪乞讨人员的救助管理，建立覆盖全市弱势群体和贫困人口的社会救助体系。

　　不断改善群众生活。争创全国社区建设示范城市，完善社区公共服务设施，促进社区科教文卫体资源共享。做好为民办实事项目。深化城镇居民住房制度改革，完善住房公积金和廉租房制度。全面推行食品质量安全准入制度，大力治理餐桌污染。完善药品、医疗器械集中招标采购制度，确保就医用药安全有效，进一步减轻群众医疗负担。完善市民服务信息系统，扩大小额支付卡使用范围，全面启用市民卡。继续解决贫困家庭学生的就学问题。加快妇女、儿童、老年人和残疾人事业发展，创建无障碍设施示范城。切实安排好城乡贫困人口的生活。

　　(六)调动各种积极因素，创造良好发展环境

　　营造开拓创新的实干氛围。增强勇于改革的锐气，鼓励敢闯敢试、敢抓敢管、敢做敢当，只要有利于最广大人民群众的利益，有利于厦门的发展，就要大胆实践，以超常规措施，冲破体制性障碍，谋求跨越式发展。保护改革者的热情，对那些想干事、能干事、干成事的干部，多一些理解、多一些支持、多一些爱护，聚集方方面面的力量，形成改革创新合力。结合市对区部分事权、财政体制和统计方式的调整，进一步理顺关系，简政放权，调动各区积极性、创造性，增强区级经济综合竞争力。

　　构筑施展才华的创业舞台。把人才作为厦门发展的第一资源，围绕发展战略，制定人才资源规划，有针对性地培养、引进、储备紧缺人才和重点人才。重视高级技工队伍的建设，鼓励发明创造，保护知识产权。全面落实柔性引进人才和居住证制度，发挥企业在用人、留人中的主体作用，实现一流人才、一流业绩、一流待遇。

　　维护安全稳定的社会环境。围绕建设“平安厦门”，深入开展社会治安综合治理，保持高压态势，对入室盗窃、街头抢夺等犯罪行为露头就打，提高群众对社会治安的满意率。严厉打击走私贩私、制假售假、偷逃骗税、商业欺诈等违法行为，严厉打击“六合彩”非法彩票和“黄、赌、毒”。加强信息安全保障工作。强化安全生产意识，完善监管体系和重特大事故应急处理预案，落实行政一把手责任追究制。加大违法建设整治力度，及时排除各种安全隐患，坚决遏制重特大事故的发生。以110联动为基础，加快建立高效统一的城市应急反应机制，增强应对各种突发事件和抵御风险的能力。重视群众信访和人民调解工作，积极预防和依法处置群体性事件。

　　三、坚持不懈建设对人民高度负责的政府

　　切实加强政府自身建设，大力弘扬求真务实精神，大兴求真务实之风，树立为民、务实、清廉、高效的政府形象。

　　狠抓落实，务求实效。强化行政首长负责制，各部门一把手对本部门工作负起总责。完善干部评议制，建立健全科学评价体系，评价一个领导不仅要看说了什么，更要看做了什么，不仅要看决策的能力，更要看落实的能力。做出的决策，要定一项做一项，项项算数，对社会、对群众作出的承诺，必须说到做到、说好做好，说得科学、做得正确，少一点表态、多一点表率，少一点形式、多一点实干。层层建立责任制，把每一项工作具体落实到每个人、每个阶段、每个项目上，坚持一具体就突破，一具体就深入，一具体就落实。带着强烈的责任抓落实，带着切实解决问题的决心抓落实，带着对基层和群众的深厚感情抓落实，从基层抓起，从基础抓起，把问题解决在基层，把各项目标和任务落到实处。建立科学规范的行政问责制，抓好责任分解、责任考核和责任追究，对每一项决策、每一个部署，及时反馈、评估和总结，经常深入第一线，看看工作进展怎样，看看决策落实的成效如何，遇到困难和阻力，要有夜不能寐、食不甘味的精神，要有一股不达目标不罢休的韧劲，一件一件抓好落实，一步一步向前推进。

　　科学决策，依法行政。认真贯彻和落实市委的各项决策。自觉接受市人大及其常委会的法律监督和工作监督，认真落实有关决议、决定。加强与政协的联系与沟通，积极支持政协履行政治协商、民主监督、参政议政的职能。认真办理人大代表议案、建议和政协提案、建议案、重大事项向人大报告并征求政协意见，推进政务公开，完善听证、公示、新闻发布、人民建议征集制度，主动接受人民群众和新闻舆论的监督，提高政务透明度和公众参与度，加强课题研究和问题调研，发挥专家学者咨询作用，促进决策的民主化和科学化。贯彻《行政许可法》，抓好行政许可项目、规定和实施机关的清理和规范工作。强化行政执法责任制，理顺执法体制，抓好市、区两级相对集中行政处罚权和综合执法，解决多头执法、重复处罚等问题。完善行政复议制度，健全政府规范性文件和重要文稿的法律审查制度，组建市长律师团，提高依法行政的能力和水平。

　　转变职能，提高效率。强化政府的社会管理和公共服务职能，改进经济调控和市场监管方式，把更多的精力放在推进社会发展和解决民生问题上。改革行政审批制度，逐步推进前置审批向后续监管的转变，从源头上提高政府工作效率。创新政府管理方式，加快市、区、镇(街)三级政务信息平台建设，推广电子政务系统应用，开展网上审批试点。结合绩效考评，加大对机关效能的奖惩力度，严肃查处“不作为”、“乱作为”现象，消除“中梗阻”，确保政令畅通。大力压缩会议、文件和评比、表彰，减少礼仪庆典，简化迎来送往，严格因公出国(境)和出差管理，集中时间和精力沉下去抓大事、抓难事。

　　艰苦奋斗，清正廉洁。全体公务员特别是领导干部要始终牢记“两个务必”，坚持奋发有力、勇挑重担，反对意志消沉、不思进取；坚持吃苦耐劳、勤俭办事，反对贪图享乐、铺张浪费。严格执行领导干部“四项纪律、八项要求”，认真落实反腐倡廉“六个机制”，强化“一岗双责”，按照“制度建设，控制源头、重在预防、前期介入”的要求，确保廉政建设各项政策规定有效执行。推进公共财政体制建设，开展国库集中支付试点，规范转移支付制度，完善预算编制和执行制度。强化预算约束力，努力降低行政成本，提高财政资金使用效益。削减一般性开支，压缩5%的部门行政经费和专项经费。严格财政财务管理，紧决查处截留挪用、滥发补贴、损失浪费等违法违规行为。加强审计监督，试行绩效预算，开展绩效审计，强化领导干部任期经济责任审计。在土地出让、工程招标、产权交易、政府采购、资金拨付、人事安排等方面严格照章办事、公开办事、按程序办事，主动接受监督检查，一旦违反，坚决查处。

　　加强学习，提高素质。适应国际一流企业不断聚集厦门、全市人民对公共服务日益增长的需要，各级政府和全体公务员必须具有全新的服务理念，提供一流的公共服务。扩大公务员竞争上岗，轮岗交流的范围和级别，使那些敢负责任、业绩突出、品德优良的公务员进入重要岗位。强化公务员培训，鼓励在职学习，支持进修深造，选派挂职锻炼，努力建设一支具备开放思维、国际视野、市场观念、法律素养的勤政善治的公务员队伍。

　　各位代表!让我们紧密团结在以胡锦涛同志为总书记的党中央周围，高举邓小平理论伟大旗帜，全面实践“三个代表”重要思想，深入贯彻党的十六大和十六届三中全会精神，在中共厦门市委的领导下，依靠全市人民，凝聚各方力量，锐意进取，埋头苦干，为再创厦门经济社会发展的新优势、新业绩而努力奋斗!
